# Emotional descriptions increase accidental harm punishment and its cortico-limbic signatures during moral judgment in autism

**DOI:** 10.1038/s41598-023-27709-x

**Published:** 2023-01-31

**Authors:** Sol Fittipaldi, Jorge L. Armony, Adolfo M. García, Joaquín Migeot, Matías Cadaveira, Agustín Ibáñez, Sandra Baez

**Affiliations:** 1 Latin American Brain Health Institute (BrainLat), Universidad Adolfo Ibáñez, Santiago, Chile; 2 Global Brain Health Institute (GBHI), University of California San Francisco (UCSF), San Francisco, USA; 3 Global Brain Health Institute (GBHI), Trinity College Dublin (TCD), Dublin, Ireland; 4 Cognitive Neuroscience Center (CNC), Universidad de San Andres, Buenos Aires, Argentina; 5 National Scientific and Technical Research Council (CONICET), Buenos Aires, Argentina; 6 Douglas Mental Health University Institute and Dept. of Psychiatry, McGill University, Montreal, Canada; 7 Departamento de Lingüística y Literatura, Facultad de Humanidades, Universidad de Santiago de Chile, Santiago, Chile; 8 Center for Social and Cognitive Neuroscience, School of Psychology (CSCN), Universidad Adolfo Ibáñez, Santiago de Chile, Chile; 9 Casa Abanico, Buenos Aires, Argentina; 10 Universidad de los Andes, Bogotá, Colombia

**Keywords:** Cognitive neuroscience, Emotion, Social neuroscience

## Abstract

Individuals with autism spectrum disorder (ASD) present difficulties in integrating mental state information in complex moral tasks. Yet, ASD research has not examined whether this process is influenced by emotions, let alone while capturing its neural bases. We investigated how language-induced emotions modulate intent-based moral judgment in ASD. In a fMRI task, 30 adults with ASD and 27 neurotypical controls read vignettes whose protagonists commit harm either accidentally or intentionally, and then decided how much punishment the protagonist deserved. Emotional content was manipulated across scenarios through the use of graphic language (designed to trigger arousing negative responses) vs. plain (just-the-facts, emotionless) language. Off-line functional connectivity correlates of task performance were also analyzed. In ASD, emotional (graphic) descriptions amplified punishment ratings of accidental harms, associated with increased activity in fronto-temporo-limbic, precentral, and postcentral/supramarginal regions (critical for emotional and empathic processes), and reduced connectivity among the orbitofrontal cortex and the angular gyrus (involved in mentalizing). Language manipulation did not influence intentional harm processing in ASD. In conclusion, in arousing and ambiguous social situations that lack intentionality clues (i.e. graphic accidental harm scenarios), individuals with ASD would misuse their emotional responses as the main source of information to guide their moral decisions. Conversely, in face of explicit harmful intentions, they would be able to compensate their socioemotional alterations and assign punishment through non-emotional pathways. Despite limitations, such as the small sample size and low ecological validity of the task, results of the present study proved reliable and have relevant theoretical and translational implications.

## Introduction

Moral judgment (the capacity to distinguish between right and wrong) arises from the interplay between reason and emotion and is critical for regulating social behavior^1–3^. Decisions about punishment of third-party harmful actions require integrating different sources of information, including emotional priors and inferences on the perpetrator’s mental state. Crucially, emotional, gut-reactions to damage and victims’ suffering are used as a source of ‘internal evidence’ to intuitively guide moral condemnation^4–8^. Highly arousing contents, such as graphic descriptions of harm, evoke increased emotional responses (e.g. disgust, contempt, sadness, stress, anguish, shock)^8–10^ which lead to harsher punishment decisions^7,11^. This emotional bias is usually found solely for intentional harms^7^, mediated by amygdala activity (involved in the processing of salient stimuli and early decoding of the purpose to harm^12^) and its connectivity with prefrontal regions (related to decision making^7,13^). Emotional responses during accidental harms are counteracted by information on the perpetrator’s innocent intentions, overruling punishment assignment irrespective of the actions’ consequences and descriptions^3,7,14^. Disentangling intentions and outcomes in moral judgment critically depends on mentalizing processes and the activity of related brain regions, such as the temporoparietal cortex^15–17^, which are markedly impaired in autism spectrum disorder (ASD)^18,19^.

Individuals with ASD present difficulties in integrating mental state information in morally conflicting tasks where intentions and outcomes are at odds^20,21^. While they are sensitive to damage and punish intentional harms as much as neurotypical (NT) people^22^, they systematically struggle in exculpating accidental harms^22–25^–but see^26,27^. This atypical moral judgment in ASD has been ascribed to diminished activity in the right temporoparietal junction^23^, a key region of the mentalizing network^28^, which has a critical role in representing others’ intentions in moral contexts^7,15–17^. Thus, in the absence of explicit information on other’s intent, persons with ASD would over-rely on actions’ negative outcomes as a source of information to guide their moral decisions. Yet, how emotionally arousing elements, such as the use of graphic language, influence this process in ASD remains unknown–let alone its neural bases–limiting the formulation of integrative theoretical and translational models.

A handful of studies has investigated the impact of emotions on ASD moral judgment, reaching controversial conclusions. In one study, elevated personal distress in ASD was associated with a reluctance to adopt utilitarian solutions in emotionally salient moral dilemmas^29^, suggesting an emotional bias on the decisions of individuals with ASD. This is in line with ASD profile of increased self-reported personal distress during socioemotional situations^30–32^, also manifested as hyperactivity in cortico-limbic regions^19,33^, which would hinder ‘rational’ moral judgments^3,29,34^. However, other works failed to show such emotional influences on ASD moral judgments^35,36^, and the only evidence on the neural bases of the processing of emotional moral situations reveals a combination of hypo- and hyperactivation in limbic (i.e. amygdala, insula, and the anterior cingulate cortex) and posterior (i.e. cingulate cortex and precuneus) regions^37^. The lack of control on mentalizing demands of the tasks used might explain inconsistent results. Indeed, persons with ASD and high-IQ might compensate their socioemotional deficits on tasks where mentalizing requirements are minimal by making use of learned rules^27,36,38–40^. Intent-based moral judgment tasks offer an adequate method to disentangle the effect of intentions and emotions and its neural bases during moral decision-making^7^, which has not been addressed in ASD.

There is a growing consensus that socioemotional atypicalities in ASD rely not on localized brain regions but on distributed networks^41,42^. Resting-state fMRI recording offers a non-invasive, easy-to-administer, and brief technique to evaluate the intrinsic coupling of functional networks (functional connectivity) independently of task performance. Such advantages make it a suitable method for biomarker investigation in clinical populations with varying levels of cognitive ability, like ASD^43,44^. Increasing evidence suggests that individuals with ASD are characterized by aberrant long-range connectivity between the medial frontal cortex and posterior regions, including the temporoparietal junction, the posterior cingulate cortex, and the precuneus^45–48^. Fronto-amygdala connections are also disrupted in ASD^49–51^. These networks have a key role in mentalizing and moral decision-making^7,13,47,52–54^. However, the resting-state correlates of moral judgment have not been investigated in ASD, precluding the investigation on novel biomarkers.

In this work, we adapted a validated fMRI task^7^ to study how emotional responses, induced by language manipulation, modulate intent-based moral judgment in adults with ASD relative to NT controls. Participants read short text-based scenarios in which a protagonist inflicts harm either accidentally or intentionally, and then decided how much punishment that person deserved. The emotional content of the vignettes was manipulated through the language used to describe harm [graphic language (GL) vs. plain language (PL)]. While both conditions featured identical amount of damage, GL descriptions were designed to trigger negative emotional responses and PL descriptions involved just-the-facts, emotionless, terms^7^. Resting-state fMRI recordings were also acquired to study off-line functional connectivity correlates of task performance. We hypothesized that, in the accidental harm condition (i.e. in the absence of explicit intentionality), GL descriptions would increase punishment severity in ASD relative to PL descriptions and NT controls’ ratings, together with enhanced cortico-limbic activations and decreased resting-state connectivity between the medial frontal cortex and posterior temporoparietal regions. Conversely, in the intentional harm condition, the effect of language manipulation on punishment ratings and the associated neural correlates would be abolished in ASD.

## Materials and methods

### Participants

We enrolled 57 Spanish-speaking adults (47 right-handed, 26 female) from clinical centers, autism associations, universities, and social network communities. Thirty participants were diagnosed with ASD by a specialized clinician (M.C.) following the Diagnostic and Statistical Manual of mental disorders (DSM-5) criteria^55^ and scoring above the cut-off (≥ 7) for either autism or ASD on the Autism Diagnostic Observation Schedule-2 (ADOS-2, module 4)^56^ (Table 1). None of them exhibited intellectual (IQ < 85) or language impairments, other primary neuropsychiatric disorder, nor substance abuse. The control group consisted of 27 NT individuals with no history of neuropsychiatric disorders or substance abuse. In line with inclusion criteria, no participant had task-fMRI contraindications, such as visual impairment, claustrophobia, metal implants, or cardiac pacemaker. Power analysis revealed that our sample size was adequate to obtain reliable effects (Supplementary Material 1).

**Table 1:** Participants’ demographic, cognitive and clinical data.

Variable	ASD group (*n* = 30)	NT group (*n* = 27)	Between-group comparison
Sex	M: 15, F: 15	M: 16, F: 11	*χ*^*2*^(1) = − 0.18, *p* = 0.66
Handedness	R: 25, L: 5	R: 22, L: 5	*χ*^*2*^(1) = 0, *p* = 0.1
Age	28.80 (6.77)	26.96 (5.68)	*t*(55) = 1.11, *p* = 0.27
Years of education	16.10 (3.27)	17.48 (3.04)	*t*(55) = − 1.65, *p* = 0.1
IQ	115.36 (10.84)	115.57 (8.19)	*t*(53) = − 0.08, *p* = 0.93
MoCA (total score)	27.03 (2.60)	27.81 (1.61)	*t*(49) = − 1.37, *p* = 0.17
IFS (total score)	24.48 (3.24)	25.37 (2.40)	*t*(53) = − 1.18, *p* = 0.24
BDI − II	14.93 (9.86)	5.81 (4.96)	*t*(43) = 4.47, *p* < 0.001
STAI (trait)	31.63 (9.99)	16.48 (7.90)	*t*(54) = 6.37, *p* < 0.001
ADOS-2 (total score)	9.27 (2.98)	–	–
ADOS-2 communication	3.3 (1.49)	–	–
ADOS-2 Reciprocal social interaction	5.97 (2.03)	–	–

Data are presented as mean (*SD*), except for sex and handedness. IQ was estimated using the vocabulary and matrix reasoning subtests from the Wechsler Abbreviated Scale of Intelligence (WASI-II). Categorical variables were analyzed via Pearson’s chi-squared test. Continuous variables were analyzed with unpaired *t* test.

*ADOS-2* autism diagnostic observation schedule-2, *AS* autism spectrum disorder, *BDI-II* beck depression inventory-II, *IFS* INECO frontal screening, *L* left, *MoCA* montreal cognitive assessment, *NT* neurotypical, *R* right, *STAI* state-trait anxiety inventory.

ASD and NT groups were matched for sex, handedness, age, years of education, and IQ (Table 1). In addition, unlike previous studies on moral judgment in ASD^22–25^, we also matched the groups in cognitive state and executive functioning (Table 1) to control for potential confounding effects^3^. Participants’ IQ was estimated using the vocabulary and matrix reasoning subtests from the Wechsler Abbreviated Scale of Intelligence (WASI-II)^57^, a widely used instrument to measure general intellectual abilities in ASD^58–60^. Cognitive state was assessed with the Montreal Cognitive Assessment (MoCA)^61^ (Supplementary Material 2.1), a brief screening tool sensitive to cognitive dysfunction in adult ASD^62,63^. Executive functions were evaluated with the INECO Frontal Screening (IFS)^64^ (Supplementary Material 2.2), a validated battery for the detection of executive-frontal dysfunction in adults with neuropsychiatric conditions^65–67^.

As expected^68^, participants with ASD showed higher depression symptoms and anxiety traits than controls, as evaluated with the Beck Depression Inventory-II (BDI-II)^69^ and the State-Trait Anxiety Inventory (STAI, trait section)^70^, respectively (Table 1). Thus, depression and anxiety scores were introduced as covariates in our main analysis.

### Ethic declarations

All participants provided written informed consent. All methods were performed in accordance with relevant guidelines and regulations from the Declaration of Helsinki. The study was approved by the ethics committee of the Institute of Cognitive Neurology (INECO) in Buenos Aires, Argentina.

### Experimental task

**Figure 1:**
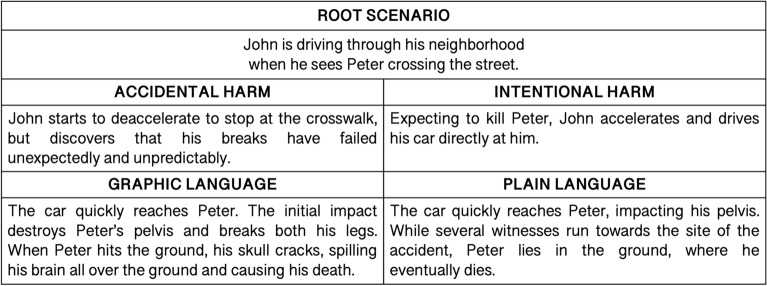
Example of experimental stimuli. From each scenario root (e.g. top row), there are four variations that differ in the intentionality of the protagonist’s action, namely, whether it is accidental or intentional (e.g. middle row), and the language used to describe harm (e.g. bottom row). Both language conditions present identical amount of damage (e.g. death). The example chosen illustrates the counterbalancing.

We employed an adapted and validated Spanish version^8^ of a moral judgment task involving punishment assignment^7^. The task consists of 24 text-based scenarios in which a protagonist (named John) harms another person or damages his/her property. Each scenario has four variations that manipulate the protagonist’s intentionality (accidental or intentional) and the type of language used to describe harm (GL or PL), resulting in four conditions: accidental/GL, accidental/PL, intentional/GL, intentional/PL. Intentionality was introduced as a within-subject factor: each participant read 12 accidental scenarios and 12 intentional scenarios, presented in pseudorandomized order. To eliminate order effects, the presentation of accidental and intentional trials was counterbalanced across participants. To avoid carry-over effects^7,71^, language was entered in the design as a between-subject factor; that is, participants assigned to the GL condition (*n*_ASD_ = 15, *n*_NT_ = 13) read only scenarios with descriptions of harm in graphic (emotional) terms, whereas participants assigned to the PL condition (*n*_ASD_ = 15, *n*_NT_ = 14) read only scenarios in plain (non-emotional) language. The GL and PL conditions were identical except for the language used to describe harm (see Fig. 1 for an example). Critically, there were not statistically significant differences between GL and PL subgroups in sex, handedness, age, years of education, IQ, cognitive state, and executive functioning (Supplementary Table S1). Also, despite the fact that participants with ASD had higher depression symptoms and anxiety traits overall (Table 1), which was controlled in the main analysis, ASD subgroups assigned to GL and PL conditions presented similar depression and anxiety scores (Supplementary Table S1). To summarize, there were four versions of each scenario: two for each language condition and, within each language condition, two versions counterbalancing accidental and intentional scenarios (Fig. 1). The number of participants presented with each version of the experimental task did not differ between groups [GL version 1: *n*_ASD_ = 9, *n*_NT_ = 6; GL version 2: *n*_ASD_ = 6, *n*_NT_ = 7; PL version 1: *n*_ASD_ = 7, *n*_NT_ = 7; PL version 2: *n*_ASD_ = 8, *n*_NT_ = 7; *χ*^2^(3) = 0.58, *p* = 0.89].

**Figure 2:**
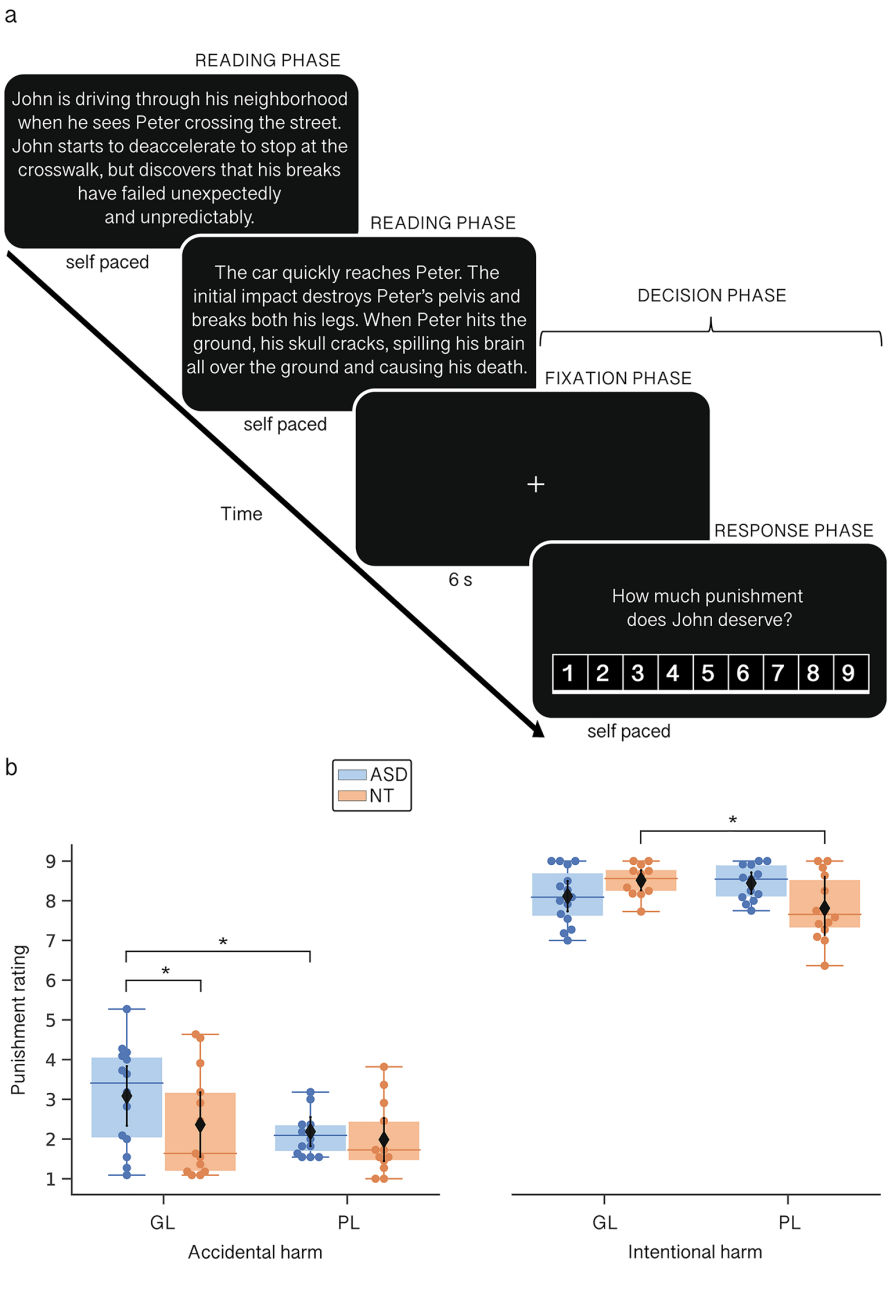
Moral judgment task. (**a**) fMRI task flow. Participants had to read scenarios describing a third-party harmful action and rate how much punishment the transgressor deserved in a Likert-scale. The figure displays the accidental harm condition described in GL (see Fig. 1 for the intentional harm/PL counterpart). Analysis of the fMRI data focused on the BOLD modulation during the fixation and response phases, collectively called ‘decision phase’. (**b**) Behavioral results. Under GL (vs. PL) descriptions, participants with ASD punished more severely the accidental harms and NT controls the intentional harms. Compared to NT controls, participants with ASD punished more the accidental harms described in GL. Only planned contrasts’ results are shown. The black dots and lines inside the boxplots indicate the mean and 95% CI respectively. *ASD* autism spectrum disorder, *GL* graphic language, *NT* neurotypical, *PL* plain language. *p < 0.05.

Following previous procedures^7^, the task was administered inside the scanner (see “Image acquisition” section). Stimuli were displayed on a screen via a projector and presented through a double mirror inserted in the head coil. We used white letters over a black background. Responses were recorded using two MRI-compatible button pads, with two buttons each. The task consisted of three phases. First, participants were instructed to read each scenario silently, at their own pace. They had to press a button with their dominant hand to move from screen to screen until the scenario was over (reading phase). Then, a fixation cross appeared in the middle of the screen for 6 s (fixation phase). Finally, participants were asked to rate how much punishment the transgressor deserved on a Likert-scale from 1 (‘no punishment’) to 9 (‘severe punishment’) (response phase). They had to use their non-dominant hand to freely move along the Likert scale, and their dominant hand to select their final response. No time limit was imposed. During the fixation phase, participants were requested to anticipate their response. Task-related fMRI analyses focused on the BOLD modulation during the fixation and response phases, collectively called ‘decision phase’, as done in the original study^7^. A schematic view of the task flow is presented in Fig. 2a. Before the scanning session, participants performed a practice trial to get familiar with the procedure.

### Behavioral data analysis

Punishment rating data were analyzed on R version 3.5.2. First, we eliminated outlier values within each participant (± 2 *SD* from the mean of each group for each intentionality and language condition). We then fitted a linear mixed-effects model using the lme4 library^72^ with intentionality (accidental and intentional), language (GL and PL), and group (ASD and NT) as fixed factors, and participant as a random effect. To control for between-group differences in depression symptoms and anxiety traits (Table 1), BDI-II and STAI-trait scores were also included in the model as covariates. The directionality of significant interaction effects was examined via planned post-hoc tests using the lsmeans library^73^. Our outcomes of interest were the effect of GL (vs. PL) on accidental and intentional harm punishment in each group separately, and group differences on punishment ratings in each intentionality and language condition. All statistical tests were two-tailed. The significance threshold was set at *p* < 0.05, uncorrected, as done in the original publication^7^, in related works on moral judgment in ASD^23,24^, and in other groups^74,75^. Effect sizes were calculated through partial eta squared (η_p_^2^) and Cohen’s *d*, when appropriate.

### Image acquisition

Image acquisition and processing steps are reported following guidelines from the Organization for Human Brain Mapping (OHBM)^76,77^. Functional images were acquired using a Philips Achieva 1.5 T scanner with a standard eight-channel head coil while participants performed the moral judgment task (see “Experimental task” section), and during 7 min rest before the experimental task. Resting-state data from one NT subject was discarded due to technical problems. A structural T1 image was also acquired for localization purposes. Acquisition parameters of each sequence are reported in Supplementary Material 3.

### Task-related fMRI data analysis

Task-related images were preprocessed using SPM12 package (https://www.fil.ion.ucl.ac.uk/spm/software/spm12/) running on MATLAB 2016a. The preprocessing pipeline followed recommendations by SPM12, as done in recent related works^78,79^ (Supplementary Material 3.1). None of the participants showed movements greater than 3 mm and/or rotations higher than 3°, and average translation and rotation parameters were similar between groups (Supplementary Table S2).

Statistical analyses were performed using a general linear model. For each participant, we modeled the onset and duration of the reading phase, the fixation phase, and the response phase for each intentionality condition (accidental and intentional scenarios). These regressors were convolved with a canonical hemodynamic response function. Six motion parameters (estimated during realignment) were included as regressors of no interest. Following the original publication^7^, our analyses focused on the BOLD signal modulation during the fixation and response phases (i.e. ‘decision phase’) (Fig. 2a). For the decision phase of each participant, we calculated contrast images for the accidental > intentional harm contrast by applying linear weights to the parameter estimates. These contrasts were then entered into a second-level group analysis. We performed a between-subject ANOVA (SPM module) with language and group as factors. The statistical threshold was set at *p* < 0.05, cluster-corrected for multiple comparisons at the whole brain level. The extent threshold was determined using AlphaSim (Rest v1.8 software)^80^ with the following parameters: individual voxel *p* < 0.005; rmm = 5; simulations = 1000. Results indicated that clusters of *k* ≥ 275 were statistically significant. Localization was derived from the Automated Anatomical Labelling Atlas (AAL)^81^. For each significant cluster, we extracted parameter estimates for each participant using the Marsbar toolbox^82^ and performed planned post-hoc tests in R. Contrasts of interests were the effect of GL on accidental and intentional scenarios in each group separately, and ASD vs. NT in the GL condition. The alpha threshold was set at *p* < 0.05, Bonferroni-corrected for multiple comparisons. Effect sizes were calculated through Cohen’s *d*. Given our small sample size, we employed the bootstrapping with replacement technique (9999 permutations) to obtain 95% confidence intervals (CI) for mean differences using the ‘boot.t.test’ function from the MKinfer package^83^.

### Resting-state fMRI data analysis

Resting-state images were preprocessed using the DPARSF V4.4 toolbox^84^ (http://rfmri.org/DPARSF) running in MATLAB 2016a. Preprocessing steps were performed following published procedures^85–90^ (Supplementary Material 3.2). None of the participants showed movements greater than 3 mm and/or rotations higher than 3º, and average translation and rotation parameters were matched between groups (Supplementary Table S3).

Functional connectivity analysis was performed following previous studies^85,88–90^. First, for each participant, the mean time course of the BOLD signal was extracted for each of the 90 regions of the AAL atlas^81^ (excluding cerebellum), by averaging the signal in all voxels comprising each region. Second, we constructed a connectivity matrix for each participant indicating the strength of association between all pairs of regions (Pearson’s correlation coefficient; DPARSF toolbox). Third, we performed a Fisher z-transformation. The resulting association scores between all pairs of regions of the AAL atlas were used to perform Spearman’s correlations with participants’ mean punishment ratings in each group (ASD and NT), intentionality (accidental and intentional harm), and language (GL and PL) condition. The alpha level was set at *p* < 0.001, uncorrected, as previously reported in studies of resting-state connectivity associations with behavior^85,88–90^.

## Results

### Behavioral results

The behavioral performance of ASD and control participants on the moral judgment task is summarized in Fig. 2b and Supplementary Table S4. In total, 5.2% of data was removed after outlier detection, evenly distributed among groups and conditions [*χ*^*2*^(3) = 0.29, *p* = 0.96]. Results from the mixed-effects model revealed a significant three-way interaction between intentionality, language, and group (*F*(1, 51) = 5.58, *p* = 0.02, η_p_^2^ = 0.1]. As hypothesized, participants with ASD punished significantly more the accidental harms when described in GL vs. PL (*p* = 0.007, *d* = 3.75), and their ratings in the GL-accidental harm condition were also significantly higher than those of NT individuals (*p* = 0.02, *d* = 3.33). Conversely, NT controls assigned more severe punishment to harmful actions described in GL (vs. PL) only when they were carried out intentionally (*p* = 0.04, *d* = 2.73), replicating original findings^7^.

No other interaction was significant, and depression (BDI-II) and anxiety (STAI-trait) scores had no significant effect on punishment ratings (Supplementary Table S5). Full mixed-model and planned post-hoc results are reported in Supplementary Table S5 and Supplementary Table S6, respectively.

### Task-related activation results

**Figure 3:**
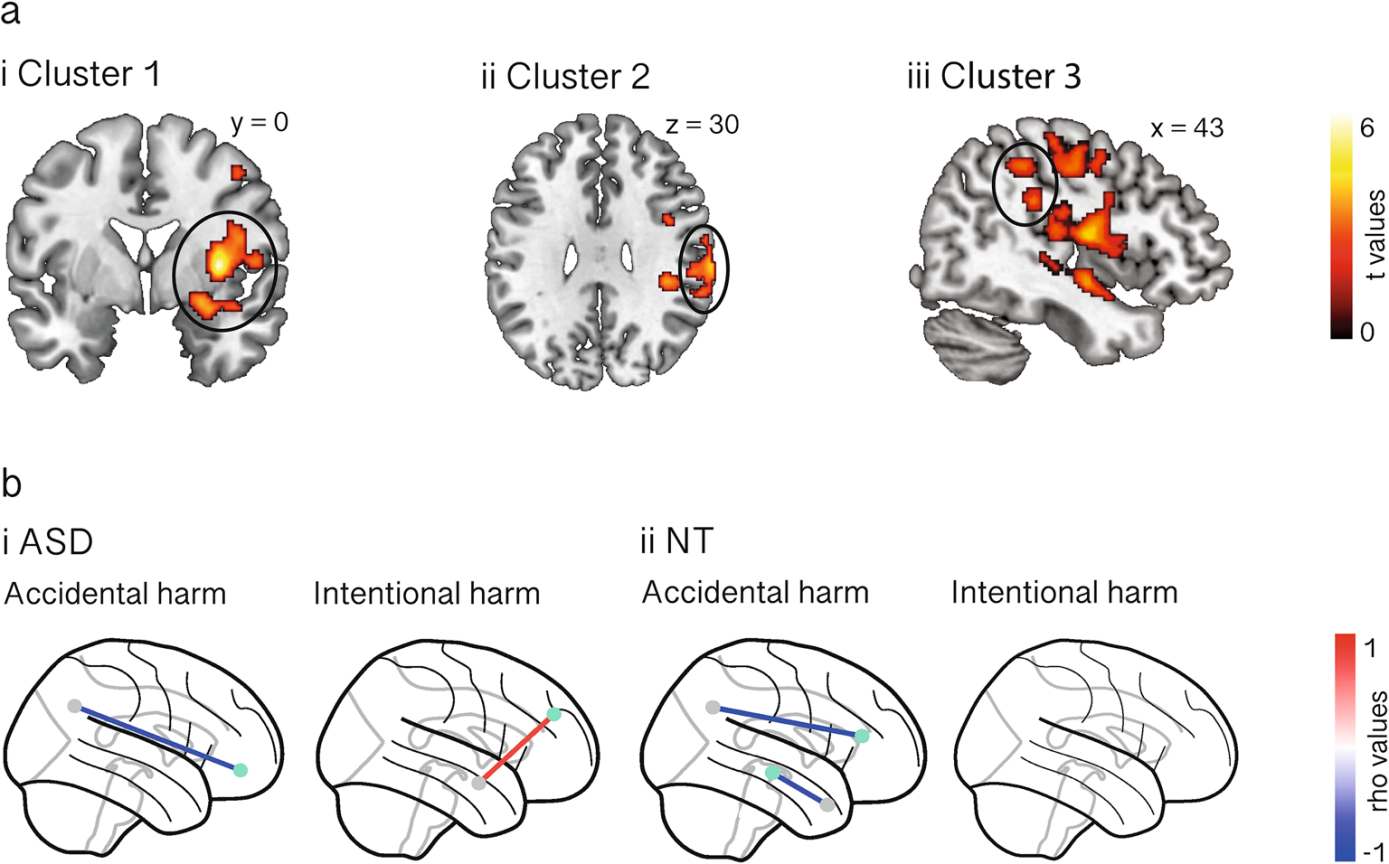
fMRI results. (**a**) Task-related results. Clusters spanning fronto-temporo-limbic (i) and precentral, postcentral/supramarginal, and posterior superior temporal (ii and iii) regions that showed a significant interaction effect between language and group (*p*_cluster-corr_ < 0.05). In the GL (vs. PL) condition, participants with ASD presented increased activation in cluster 1 for the accidental > intentional harm contrast (*p*_Bonferroni-corr_ = 0.04), and NT controls presented increased activation in the three clusters for the intentional > accidental harm contrast (all *ps*_Bonferroni-corr_ ≤ 0.001). Compared to NT controls, participants with ASD presented increased activation in the three clusters in the GL-accidental harm condition (all *ps*_Bonferroni-corr_ < 0.05). Contrast maps were created using SPM12 (https://www.fil.ion.ucl.ac.uk/spm/software/spm12/) and plotted in MRIcron (V1.0.20190902, https://www.nitrc.org/projects/mricron). Images are displayed in neurological convention. (**b**) Resting-state results. Functional connectivity associations with punishment ratings in the GL condition for ASD (i) and NT control (ii) groups. Punishment of accidental harms was negatively associated with medial prefrontal-angular gyrus connectivity in both groups, while punishment of intentional harms was positively associated with fronto-amygdala connectivity only in the ASD group (all *ps*_unc_ < 0.001). Images were created using the Nilearn library for Python (V0.9.2, https://nilearn.github.io/stable/index.html). *ASD* autism spectrum disorder, *NT* neurotypical.

Analysis of the fMRI data revealed a significant interaction (*p*_cluster-corr_ < 0.05) between language and group in a large right fronto-temporo-limbic cluster spanning the inferior frontal gyrus, the superior temporal gyrus and temporal pole, the amygdala, and the insula and adjacent Rolandic operculum (Fig. 3ai and Supplementary Table S7), and in two additional right clusters involving precentral, postcentral/supramarginal, and posterior superior temporal regions (Fig. 3aii and iii, and Supplementary Table S7). Post-hoc analysis on parameter estimates revealed that, in the GL (vs. PL) condition, participants with ASD presented increased activation in cluster 1 for the accidental > intentional harm contrast (*p*_Bonferroni-corr_ = 0.04, *d* = 3.51, 95% CI  0.07–0.39), while NT controls showed increased activation in the three clusters for the intentional > accidental harm contrast (cluster 1: *p*_Bonferroni-corr_ < 0.001, *d* = 7.74, 95% CI  0.34–0.73; cluster 2: *p*_Bonferroni-corr_ < 0.001, *d* = 6.51, 95% CI  0.39–1.07; cluster 3: *p*_Bonferroni-corr_ = 0.001, *d* = 5.63, 95% CI  0.27–0.76). Between-group comparisons showed that, in the GL-accidental harm condition, participants with ASD presented increased activation than NT controls in the three clusters (cluster 1: *p*_Bonferroni-corr_ = 0.007, *d* = 4.48, 95% CI  0.14–0.48; cluster 2: *p*_Bonferroni-corr_ = 0.01, *d* = 4.25, 95% CI  0.11–0.83; cluster 3: *p*_Bonferroni-corr_ = 0.02, *d* = 3.94, 95% CI  0.13–0.83) (See details in Supplementary Table S7).

### Resting-state functional connectivity results

The more participants with ASD punished accidental harms in the GL condition, the lower their functional connectivity between the left orbitofrontal cortex and the left angular gyrus (*n* = 14, Spearman’s *rho* = − 0.81, *p*_unc_ < 0.001) (Fig. 3bi, left). In addition, in the ASD group, higher punishment of intentional harms in the GL condition was associated with greater functional connectivity between the left superior medial frontal cortex and the left amygdala (*n* = 15, Spearman’s *rho* = 0.80, *p*_unc_ < 0.001) (Fig. 3bi, right). There were no significant associations between ASD punishment ratings of accidental or intentional harms described in PL and their functional connectivity.

In the NT group, higher punishment of accidental harms in the GL condition was associated with lower functional connectivity between the right anterior cingulate cortex and the left angular gyrus (*n* = 13, Spearman’s *rho* = − 0.88, *p*_unc_ < 0.001), and between the right temporal pole and the left hippocampus (*n* = 13, Spearman’s *rho* = − 0.83, *p*_unc_ < 0.001) (Fig. 3bii, left). No significant functional connectivity associations emerged for PL descriptions of accidental harms, and for either GL or PL descriptions of intentional harms in the NT sample (Fig. 3bii, right).

## Discussion

To our knowledge, this is the first study addressing the influence of emotions on intent-based moral judgment in ASD and its neural signatures. As hypothesized, in the ASD group, emotional (GL) descriptions increased punishment of accidental harms, in association with enhanced cortico-limbic activation and diminished resting-state connectivity between the medial frontal cortex and posterior temporoparietal regions. This effect was not present in intentional harm scenarios, suggesting differential behavioral and neural response patterns according to the information on the transgressor’s mental state. These results have relevant theoretical and clinical implications, as described below.

Previous evidence has consistently shown that individuals with ASD fail to forgive accidental harms given core difficulties in representing others’ innocent intentions together with an over-reliance on actions’ negative outcomes^23,24^. Our results extend this interpretation by revealing a key role of emotional responses in this process. In the present study, participants with ASD assigned more severe punishment to accidental harms when described in emotional (graphic) vs. plain (just-the-facts, emotionless) terms despite both conditions featuring identical amount of damage. Their punishment ratings of accidental harms were also higher than those of NT controls in the GL (but not PL) condition. Taken together, in the absence of explicit intentionality clues, individuals with ASD would misuse their emotional responses (beyond outcomes, as implied by the lack of between-group differences in PL condition) to guide their punishment decisions.

Task-related fMRI results support the above interpretation. Emotional (GL) descriptions of accidental harms elicited increased activation in the inferior frontal gyrus, the superior temporal gyrus and temporal pole, the amygdala, and the insular/opercular region in the ASD group relative to PL descriptions. In addition, increased activations for accidental harms described in GL were found across precentral, postcentral/supramarginal, and posterior superior temporal cortices in ASD in comparison to the NT control group. The temporal, postcentral/supramarginal, and limbic (amygdala, insula) regions found here overactivated in ASD are typically involved in emotional processes and the emotional dimension of empathy^91–94^. In addition, in light of previous reports^95,96^ the overactivation of the inferior frontal gyrus and the precentral cortex in ASD suggests a dysfunction in the mirror neuron system, a subset of neurons that fire when performing an action and when seeing another person performing the same action^97^, which has been proposed as a neural substrate of certain domains of empathy^98^. In this line, recent evidence shows that ASD presents exacerbated reactivity to others’ suffering^19,29,30,32,33,99,100^. More particularly, according to the ‘empathy imbalance hypothesis of autism’, the profile of ASD is characterized by heighten emotional empathy (related to emotional arousal) together with low cognitive empathy (related to mentalizing)^30^. Deficits in emotion regulation might also be inherent of ASD^101–103^. Thus, in arousing and ambiguous social situations (i.e. GL-accidental harm scenarios), the emotional hyperreactivity together with deficits in mental state understanding would prevent individuals with ASD to override biases in their moral decisions, as NT usually do in such situations.

Convergently, in participants with ASD, the severity of punishment assigned to accidental harms in the emotional (GL) condition was associated with decreased resting-state functional connectivity between the left orbitofrontal cortex and the left angular gyrus. Similar results were found for NT controls, suggesting a dimensional mechanism^104–106^. The medial/ventral parts of the prefrontal cortex and the angular gyrus in the temporoparietal junction are core hubs of the default mode network that subserves mentalizing abilities^53,54^, and altered connectivity within this network is a common finding in persons with ASD^48,107^, in relation to symptom severity^47^. Arguably, weakened default mode network connectivity would represent less resources to integrate information on others’ innocent intentions, hindering the ability to counteract salient emotional information in a flexible manner. In support of this claim, in NT subjects, a medial prefrontal-temporoparietal circuit suppresses amygdala activity during emotional (GL) descriptions of accidental harms, preventing punishment assignment^7^. Future studies should test whether default mode network-mediated mentalizing deficits explain atypicalities in emotion-guided moral judgment in ASD.

Unlike NT controls, GL descriptions had no effect on punishment assignment to intentional harms and did not induce active brain modulations in ASD. On the other hand, there were not between-group differences in punishment ratings for intentional harms, as shown in previous research^22^. Thus, while being able to punish intentional harms, individuals with ASD would not exhibit the typical intuitive-emotional bias shown by NT persons in this condition^7^. In contrast, they would profit from the explicit information on the perpetrator’s mental state to assign punishment through non-emotional pathways by employing compensation strategies, possibly based on the use of learned social rules^27,36,38–40^. Multiple neurocognitive mechanisms might facilitate such compensation in ASD^39^, including high intellectual abilities, preserved executive functions, the recruitment of additional brain networks (e.g. hippocampal-memory regions^108^), and/or a combination of some of them. Novel experimental tasks should be designed to underscore the specific compensation strategies that persons with ASD display while making moral judgments.

Punishment ratings of participants with ASD in the GL-intentional harm condition were associated with increased resting-state functional connectivity between the left superior medial frontal cortex and the left amygdala. The lack of such association in the NT group could be due to low response variability. Fronto-amygdala connections are critical for emotional-cognitive integration in moral judgment^13,52^ and gut-driven punishment decisions^7,13^. The amygdala plays a role in rapidly reacting to intended harm and guiding decision-making in a bottom-up manner by sending inputs to the prefrontal cortex^109^. Individuals with ASD are characterized by aberrant fronto-amygdala connectivity during socioemotional processing and at rest^50,51^. We speculate that a stronger connectivity among those regions facilitates a greater use of emotions to inform decision-making in salient moral situations.

Interestingly, while task-related results were lateralized to the right hemisphere, resting-state functional connectivity results mainly involved regions in the left hemisphere. In coherence with our results, previous fMRI activation findings on moral judgment in ASD have highlight modulations in right regions (e.g. right temporoparietal junction^23^). However, and also consistently with our results, connectivity associations of the current task in healthy participants have engaged predominantly left regions (left amygdala, left prefrontal cortex, and left temporoparietal junction^7^). Further research could test the hypothesis that the right hemisphere has a prominent role on ‘in-vivo’ emotional responses while the left hemisphere participates more in bottom-up emotional decision-making and top-down emotion regulation.

Our results have several implications. On the theoretical side, the findings reported here provide novel evidence on how emotional responses and mental state inference interact to drive atypical moral judgment in ASD. While previous research has addressed the impact of emotions on moral judgment in ASD, suggesting both emotional biases^29^ and rule-based response strategies^36^ at the basis of participants’ atypicalities, these tasks did not control for mentalizing demands. Our experimental design allowed, for the first time, to disentangle how emotional content and intentionality influence moral judgment in ASD. Also, our study is the first in addressing the active and resting-state neural correlates of emotion-driven intent-based moral judgment in ASD, offering new insights on potential explanatory mechanisms. On the clinical side, our results pave the way to better understand socioemotional difficulties in ASD (that can sometimes be very subtle) and explore new non-pharmacological and pharmacological interventions and dimensional biomarkers. For instance, emotion regulation strategies (e.g. reappraisal)^110,111^ and intranasal oxytocin administration^112^ can attenuate limbic-amygdala activity. Then, these interventions might also impact moral decisions and (potentially) moral behaviors in ASD.

Some limitations and further research must be discussed. First, our sample size was small. Although an a priori power analysis confirmed its adequacy for our statistical design (Supplementary Material 1), we acknowledge that, given that scenarios’ language is a between-subject factor, GL and PL groups were composed by a low number of participants (*n* = 15). However, this sample size is similar to the original publication^7^. In addition, participants with ASD were evaluated by an expert clinician following standardized criteria (DSM-5^55^) and their scores in the ADOS-2 scale^56^ (the gold-standard instrument for ASD diagnosis) were consistent with those reported in validation studies^113,114^. Thus, we have no reason to believe our ASD sample is not representative of the corresponding population. Moreover, behavioral results in the ASD group were consistent with predictions.

Second, we used a 1.5 T scanner while now 3 T scanners are the standard in the field. Increased magnetic strength provide significantly higher signal-to-noise ratio and sensitivity to BOLD contrast^115,116^. However, we performed fMRI analysis and report significant results following standard neuroimaging practices^76,77^, including cluster-correction and reduced number of hypothesis-driven Bonferroni-corrected post-hoc tests^117^. We also report effect sizes and CI, which are superior than *p*-values to gauge the plausibility of a given result^117^. CI were obtained through bootstrapping with 9999 permutations as detailed elsewhere^83^. Permutation tests are suitable for small samples and do not rely on assumptions about the data distribution^118^, suggesting that our results are unlikely driven by extreme observations. Also, as our behavioral results, fMRI effects in the NT sample are in line with the original publication featuring increased cortico-limbic activations during the processing of intentional harms described in GL^7^. In addition, higher magnetic field strength might not always provide superior fMRI results in social cognition studies since susceptibility artifacts are greater and can have adverse effects in the detection of activity in critical regions such as the amygdala and potentially others, including the anterior hippocampus, the anterior temporal pole, and the inferior orbitofrontal cortex^116^. Finally, not only classical studies on the neural bases of moral cognition have been carried out using 1.5 T scanners^119–121^, but also recent ones^122,123^. In sum, while we cannot rule out potential false negative results, our statistical approach to fMRI analysis (corrections for multiple comparisons and bootstrapping) controlled for false positive findings. With the increased availability of ultra-high-field fMRI in cognitive neuroscience arena^124,125^, future studies should replicate our results and explore more subtle activations.

Third, the task used featured extreme life-or-death scenarios, which unlikely represent the kind of situations people encounter on a daily basis when making moral decisions. The use of serious transgressions (as well as sacrificial dilemmas) to study moral judgment has been criticized for the lack of external validity, psychological realism, and personal relevance^126,127^. Despite this limitation, such paradigms are standard procedures in the field, as revealed by their extended use in ASD^21,22^ and other neuropsychiatric conditions^1^, which facilitates the comparability of findings. Our choice of the current task is further grounded on the following reasons: (a) it allows to disentangle emotions and intentionality influences on moral judgment, (b) is associated with reliable fMRI correlates^7^, and (c) has proven suitable to study moral decisions in Spanish-speaking populations^8^. In any case, future studies should employ more ecological designs to increase our understanding of everyday moral judgment in ASD. For instance, Bellesi et al.^25^ have developed novel tasks to assess how individuals with ASD process transgressions of moral rules in more familiar situations (e.g. lying about owns’ skills in a job interview). In the same line, Callenmark et al.^128^ have used naturalistic vignettes to evaluate how participants with ASD depict social norms in explicit and implicit ways. More research is needed unravel the neural correlates of this kind of realistic moral judgment.

Fourth, following the original publication^7^, our fMRI analysis focused on the ‘decision phase’ of the experimental task, excluding the reading phase. However, given that text processing might differ between ASD and NT people, as an exploratory strategy, we re-run the fMRI analysis using the reading phase as input (Supplementary Material 4). No significant interaction results were found. This is not surprising since the task was designed to maximize the detection of relevant BOLD effects surrounding the participant’s response^7,129,130^, not during reading. Moreover, reading might involve other components unrelated to moral decision-making, with the potential to mask effects of interest. Future studies employing other techniques with greater temporal resolution (such as electroencephalography) or ultra-high-field fMRI should address the time-course of moral decision-making and perform group comparisons to better understand underlying processes. Relatedly, while we cannot ensure that participants actually paid attention while reading, we have shown comparable reaction times across groups, which indirectly suggests the deployment of equivalent processing resources (Supplementary Material 3.1).

Fifth, we did not compare resting-state functional connectivity patterns between ASD and NT control groups because it was beyond the scope of the present study. This issue has been extensively investigated and summarized elsewhere^48,131^. Here, we were interested in exploring brain connectivity associations with behavior, following previous methodologies^85,88–90^. In any case, further research focused on ASD intrinsic brain dynamic may assess basic differences across multiple networks in comparison with NT controls.

Finally, we did not include independent measures of mentalizing, empathy, and/or alexithymia to assess the potential moderating effect of those relevant variables in our results (see^29,36^ for alexithymia effects on ASD moral judgment). Future studies should address these limitations and evaluate the impact of emotions and moral judgment on functionality and social behavior of persons with ASD in real life.

In conclusion, we revealed a key role of emotional content in driving ASD atypical intent-based moral judgment, supported by convergent behavioral, active, and resting-state fMRI evidence. Our results suggest that effects of emotional descriptions on the moral decisions of individuals with ASD are evident in situations where there are not explicit intentionality clues. Taken together, these findings open a new avenue to develop translational models and treatment strategies for emotionally guided moral decisions in ASD.

## Supplementary Information

Supplementary Information.

## Data Availability

Data that support the findings of this study are available online at https://bit.ly/3DM70Iw.
