# Supplementary material for: Emotional descriptions increase accidental harm punishment and its cortico-limbic signatures during moral judgment in autism
Source: Sci Rep. 2023 Jan 31;13:1745. doi: 10.1038/s41598-023-27709-x (PMC9889714; doi:10.1038/s41598-023-27709-x)
Supplement: Supplementary file 1 — Supplementary Information. [file 41598_2023_27709_MOESM1_ESM.pdf]

Supplementary Material

Emotional descriptions increase accidental harm punishment and its cortico-limbic signatures during moral judgment in autism

Running title: Emotions and moral judgment in autism

Sol Fittipaldi[1,2,3,4], Jorge L. Armony[5], Adolfo M. García[2,3,4,6], Joaquín Migeot[1,7], Matías Cadaveira[8], Agustín Ibáñez[1,2,3,4], & Sandra Baez[9]*

[1] Latin American Brain Health Institute (BrainLat), Universidad Adolfo Ibáñez, Santiago, Chile

[2] Global Brain Health Institute (GBHI), University of California San Francisco (UCSF), USA, and Trinity College Dublin (TCD), Ireland

[3] Cognitive Neuroscience Center (CNC), Universidad de San Andres, Buenos Aires, Argentina

[4] National Scientific and Technical Research Council (CONICET), Buenos Aires, Argentina

[5] Douglas Mental Health University Institute and Dept. of Psychiatry, McGill University, Montreal, Canada

[6] Departamento de Lingüística y Literatura, Facultad de Humanidades, Universidad de Santiago de Chile, Santiago, Chile

[7] Center for Social and Cognitive Neuroscience, School of Psychology (CSCN), Universidad Adolfo Ibáñez, Santiago de Chile, Chile

[8] Casa Abanico, Buenos Aires, Argentina

[9] Universidad de los Andes, Bogotá, Colombia

*Corresponding author: Sandra Baez, MS, PhD. Universidad de los Andes, Phone: +571 3394999 Ext.: 5560, Fax: +57(1) 3324539, Cra. 1 #18a-12, Bogotá, Colombia. Email: sj.baez@uniandes.edu.co

1. Sample size estimation

Given our statistical design (2x2x2 mixed ANOVA, within-between interaction), a power analysis run on G*Power 3.1[1] showed that a sample size of 48 participants was required to achieve an effect size of 0.25 (as a previous study with this paradigm[2]) with $\alpha = 0.05$, and a power of 80%. A post-hoc power estimation confirmed that this assumption was met, since our analyses were performed on 57 participants, yielding a power of 0.87.

2. Cognitive assessment

2.1. The Montreal Cognitive Assessment

The Montreal Cognitive Assessment (MoCA)[3] is a brief screening tool that includes fourteen subtests to tap the following domains: attention, visuospatial, visuoconstructional and executive functions, language, naming, memory, abstraction, and orientation. Its maximum score is 30 (cut-off: 26), with higher scores indicating better performance.

2.2. The INECO Frontal Screening

The INECO Frontal Screening (IFS) battery[4] includes eight subtests to assess three executive functions: response inhibition and set shifting, working memory, and abstraction capacity. The IFS maximum score is 30 (cut-off: 25), with higher scores indicating better performance.

3. Image acquisition and preprocessing

3.1. Task-related fMRI data

While participants performed the experimental task, we acquired GRE-EPI volumes in sequential ascent, parallel to the anterior-posterior commissures, covering the whole-brain except the cerebellum. The following parameters were used: TR = 2000 ms; TE = 50 ms; flip angle = 90°; nº of slices = 20; matrix dimension = 64 × 64; voxel size in plane = 3.75 × 3.75 mm; slice thickness = 5 mm; interslice gap = 0 mm; sequence duration = 25 min; total number of volumes = 750. The run began with five dummy volumes (subsequently discarded from analyses) to allow for equilibration effects. After the fifth volume, the task was automatically triggered.

Before preprocessing, for each participant, we segmented the volumes corresponding to the task from the complete fMRI series (750 volumes in total). The remaining volumes were discarded. The ASD group took an average of 555.66 volumes (*SD* = 117) to perform the task, and the NT group an average of 579.85 (*SD* = 83.74). The difference was not statistically significant [$t(52) = -0.90$, $p = 0.3$], revealing that both groups required comparable time to complete the task. In

addition, reaction times during the reading phase were similar between groups [M_{ASD} = 37.27 s, SD_{ASD} = 14.41; M_{NT} = 37.88 s, SD_{NT} = 6.77; $t(42) = 0.2$, $p = 0.83$].

As recommended by SPM12, and done in recent related works[5,6], preprocessing steps included manual reorientation of each participant's scan series to the anterior-posterior commissures, slice timing correction using the middle slice of each volume as reference scan, realignment and unwrapping to correct for movement artefacts, co-registration with each participant's T1 structural image (after tissue segmentation), normalization to the MNI space employing the echo-planar imaging (EPI) template with an isotropic 2 mm voxel size to correct for inter-subject spatial variability, and smoothing using an 8 mm Gaussian kernel (full-width at half-maximum) to improve the signal-to-noise ratio.

3.2. Resting-state fMRI data

We obtained resting-state fMRI recordings from 56 participants. GRE-EPI volumes were acquired in a sequentially ascending order, parallel to the anterior-posterior commissures, covering the whole brain except the cerebellum. The following parameters were used: TR = 2000 ms; TE = 50 ms; flip angle = 90°; nº of slices = 22; matrix dimension = 64 × 64; voxel size in plane = 3.75 × 3.75 mm; slice thickness = 5 mm; interslice gap = 0 mm; sequence duration = 7 min; total number of volumes = 210. Participants were requested to lie still with their eyes open (to prevent them falling asleep[7]) and not to think about anything in particular.

Before preprocessing, the first 10 volumes of each participant's series were discarded to ensure that magnetization achieved a steady state. Then, following previous procedures[8-13] volumes were manually reoriented the to the anterior-posterior commissures, slice-timing corrected (using the middle slice of each volume as the reference scan), realigned to the first scan of the session to correct head movement (SPM functions, called by DPARSF), normalized to the MNI space using the EPI template from SPM, smoothed using an 8 mm full-width-at-half-maximum isotropic Gaussian kernel (SPM functions, called by DPARSF), and filtered (0.01-0.08 Hz). Six motion parameters, CFS, and WM signals were regressed out to reduce the effect of motion and physiological artifacts such as cardiac and respiration effects (REST V1.7 toolbox, called by DPARSF). Motion parameters were estimated during realignment. CFS and WM masks were derived from the tissue segmentation of each participant's T1 scan in native space using SPM12 (after co-registration of each participant's structural image with the functional image).

3.3. Anatomical MRI data

For localization purposes, for each subject, we acquired a T1-weighted anatomical 3D spin echo sequence parallel to the plane connecting the anterior and posterior commissures, covering the whole brain. The following parameters were used: TR = 7489 ms, TE = 3420 ms, flip angle = 8º, nº of slices = 196, matrix dimension = 256 × 240, voxel size = 1 × 1 × 1 mm, interslice gap = 0 mm, total scan duration = 7 min.

4. fMRI analysis of the reading phase

Given that text processing might differ between ASD and NT people, as a complementary analysis, we re-run the fMRI ANOVA using the reading phase of each participant as input instead of the ‘decision phase’. At the first level, contrast images were calculated for the accidental > intentional harm contrast by applying linear weights to the parameter estimates. At the second-level group analysis, we performed a between-subject ANOVA (SPM module) with language and group as factors. No interaction results survived when controlling for multiple comparisons at the cluster level ($p < 0.05$, corrected for multiple comparisons using AlphaSim, $k \geq 203$). Thus, post-hoc group comparisons were not performed.

Supplementary Tables

Table S1. Participants' demographic, cognitive, and clinical data according to task condition

Variable	GL ASD group (n = 15)	GL NT group (n = 13)	PL ASD group (n = 15)	PL NT group (n = 14)	Between-group comparison
Sex	M: 8, F:7	M: 9, F: 4	M: 7, F: 8	M: 7, F: 7	$\chi^2(3) = 1.63, p = 0.65$
Handedness	R: 11, L: 4	R: 10, L: 3	R: 14, L: 1	R: 12, L: 2	$\chi^2(3) = 2.46, p = 0.48$
Age	26.93 (6.5)	25.53 (4.37)	30.66 (6.72)	28.28 (6.56)	$F(3, 53) = 1.78, p = 0.16$
Years of education	14.86 (2.41)	17.15 (2.57)	17.33 (3.61)	17.78 (3.49)	$F(3, 53) = 2.64, p = 0.06$
IQ	112.73 (9.55)	116.83 (9.66)	118 (11.71)	114.5 (6.88)	$F(3, 52) = 0.87, p = 0.46$
MoCA (total score)	26.8 (3.02)	28 (1.29)	27.26 (2.18)	27.64 (1.9)	$F(3, 53) = 0.75, p = 0.52$
IFS (total score)	23.53 (3.45)	25.61 (2.87)	25.43 (2.8)	25.14 (1.94)	$F(3, 53) = 1.64, p = 0.18$
BDI-II	17.73 (11.52)	6.53 (5.62)	12.13 (7.21)	5.14 (4.36)	$F(3, 53) = 7.85, p < 0.001$<br>GL-ASD vs. GL-NT: $p = 0.002$<br>GL-ASD vs. PL-ASD: $p = 0.21$<br>GL-ASD vs. PL-NT: $p = 0.0004$<br>PL-ASD vs. GL-NT: $p = 0.24$<br>PL-ASD vs. PL-NT: $p = 0.08$<br>PL-NT vs. GL-NT : $p = 0.96$
STAI (trait)	31.26 (9.93)	18.15 (8.43)	32 (10.39)	14.92 (7.34)	$F(3, 53) = 13.25, p < 0.001$<br>GL-ASD vs. GL-NT: $p = 0.002$<br>GL-ASD vs. PL-ASD: $p = 0.99$<br>GL-ASD vs. PL-NT: $p = 0.0001$

					PL-ASD vs. GL-NT: $p = 0.001$<br>PL-ASD vs. PL-NT: $p < 0.0001$<br>PL-NT vs. GL-NT: $p = 0.79$
ADOS-2 (total score)	8.46 (2.55)	------	10.06 (3.23)	------	$t(26) = -1.5, = 0.14$
ADOS-2 Communication	3 (1.13)	------	3.6 (1.76)	------	$t(23) = -1.1, = 0.27$
ADOS-2 Reciprocal social interaction	5.46 (1.92)	------	6.46 (2.06)	------	$t(27) = -1.37, = 0.18$

Data are presented as mean (*SD*), except for sex and handedness. IQ was estimated using the vocabulary and matrix reasoning subtests from the Wechsler Abbreviated Scale of Intelligence (WASI-II). Categorical variables were analyzed via Pearson's chi-squared test. Continuous variables were analyzed with ANOVAs or unpaired *t*-test, when appropriate. Follow-up post-hoc tests are corrected for multiple comparisons using the Tukey HSD method. ADOS-2: Autism Diagnostic Observation Schedule-2; ASD: Autism spectrum disorder; BDI-II: Beck Depression Inventory-II; GL: graphic language; IFS: INECO Frontal Screening; L: left; MoCA: Montreal Cognitive Assessment; NT: neurotypical; PL: plain language; R: right; STAI: State-Trait Anxiety Inventory.

Table S2. Motion parameters during the task-fMRI sequence

Variable	ASD group ($n = 30$)	NT group ($n = 27$)	Between-group comparison
Average translation	0.08 (0.06)	0.06 (0.03)	$t(43) = 1.58, p = 0.11$
Average rotation	0.08 (0.09)	0.05 (0.03)	$t(38) = 1.74, p = 0.08$

Data are presented as mean (*SD*). Variables were analyzed with unpaired *t*-test. ASD: Autism spectrum disorder; NT: neurotypical.

Table S3. Motion parameters during the resting-state fMRI sequence

Variable	ASD group ($n = 30$)	NT group ($n = 27$)	Between-group comparison
Average translation	0.05 (0.04)	0.06 (0.02)	$t(47) = -0.07, p = 0.93$

Average rotation	0.05 (0.05)	0.04 (0.02)	$t(41) = 0.75, p = 0.45$

Data are presented as mean (*SD*). Variables were analyzed with unpaired *t*-test. ASD: Autism spectrum disorder; NT: neurotypical.

Table S4. Participants' performance on the moral judgment task

Condition		Punishment rating	
		ASD group	NT group
Accidental harm	GL	3.08 (1.29)<br>$n = 14/15$	2.36 (1.33)<br>$n = 13/13$
	PL	2.18 (0.63)<br>$n = 14/15$	1.98 (0.89)<br>$n = 13/14$
Intentional harm	GL	8.11 (0.68)<br>$n = 15/15$	8.51 (0.39)<br>$n = 12/13$
	PL	8.44 (0.44)<br>$n = 13/15$	7.81 (0.81)<br>$n = 14/14$

Data are presented as mean (*SD*). *n* indicates the sample size for each group corresponding to each condition after outlier removal (e.g., In the ASD group, 15 participants read GL descriptions and 15 participants read PL descriptions, as language is a between-subject factor. In the accidental harm condition, one outlier datapoint was removed in each language condition, resulting in $n = 14$ subjects per group). ASD: Autism spectrum disorder; GL: graphic language; NT: neurotypical; PL: plain language.

Table S5. Mixed-effects model results

	DF	*F*-value	*p*-value	η_p^2
Intentionality	1, 51	1253.42	< 0.001	0.96
Language	1, 50	5.84	0.01	0.1
Group	1, 51	2.87	0.09	0.05

BDI-II	1, 49	0.31	0.57	0.006
STAI-trait	1, 54	0.03	0.85	0.001
Intentionality*language	1, 51	1.97	0.16	0.04
Intentionality*group	1, 51	1.05	0.30	0.02
Language*group	1, 51	0.38	0.53	0.007
Intentionality*language*group	1, 51	5.58	0.02	0.10

BDI-II: Beck Depression Inventory-II; STAI: State-Trait Anxiety Inventory.

Table S6. Planned post-hoc tests for intentionality by language by group interaction

**Contrast**		***t***	***p*-value***	**Cohen's *d***
**GL vs. PL on accidental harm**	ASD	2.72	0.007	3.75
	NT	1.14	0.25	1.54
**GL vs. PL on intentional harm**	ASD	-0.83	0.4	1.12
	NT	2.04	0.04	2.73
**ASD vs. NT**	GL accidental harm	2.28	0.02	3.33
	GL intentional harm	-0.72	0.47	1.06
	PL accidental harm	0.77	0.44	1.16
	PL intentional harm	1.87	0.06	2.79

*Uncorrected. ASD: Autism spectrum disorder; GL: graphic language; NT: neurotypical; PL: plain language.

Table S7. Interaction results on fMRI activation for the accidental > intentional harm contrast

	N° voxels	Peak *t*	Peak *p*-value*	MNI Coordinates			Region	Post-hoc results**
				X	Y	Z		
Cluster 1	1560	5.96	< 0.001	34	0	6	R Putamen	**GL vs. PL:**<br>**ASD:** $t(53) = 2.48$; $p = 0.04$; $d = 3.51$, 95% CI = 0.07 – 0.39<br>**NT:** $t(53) = -5.39$; $p < 0.001$; $d = 7.74$, 95% CI = -0.73 – -0.34<br>**ASD vs. NT in GL:** $t(53) = 3.17$; $p = 0.007$; $d = 4.48$, 95% CI = 0.14 – 0.48
		5.02	< 0.001	34	6	12	R Insula	
		4.47	< 0.001	44	-6	14	R Rolandic operculum	
		3.83	< 0.001	26	0	-12	R Amygdala	
		3.71	< 0.001	50	8	-10	R Superior temporal pole	
		3.57	< 0.001	54	-20	2	R Superior temporal	
		3.68	< 0.001	34	18	22	R Inferior frontal	
		3.32	< 0.001	44	-20	14	R Heschl	
Cluster 2	806	4.07	< 0.001	64	-22	30	R Supramarginal	**GL vs. PL:**<br>**ASD:** $t(53) = 1.12$; $p = 0.8$; $d = 1.58$, 95% CI = -0.07 – 0.41<br>**NT:** $t(53) = -4.62$; $p < 0.001$; $d = 6.51$, 95% CI = -1.07 – -0.39<br>**ASD vs. NT in GL:** $t(53) = 3.01$; $p = 0.01$; $d = 4.25$, 95% CI = 0.11 – 0.83
		3.43	< 0.001	42	-12	46	R Precentral	
		3.43	< 0.001	46	-22	56	R Postcentral	
		3.07	< 0.001	60	-38	22	R Superior temporal	
Cluster 3	328	3.67	< 0.001	38	-34	46	R Postcentral	**GL vs. PL:**<br>**ASD:** $t(53) = 1.75$; $p = 0.25$; $d = 2.48$, 95% CI = -

							0.01 – 0.44
	3.50	< 0.001	36	-34	42	R Supramarginal	**NT:** $t(53) = -5.47$; $p = 0.001$; $d = 5.63$, 95% CI = -0.76 – -0.27<br>**ASD vs. NT in GL:** $t(53) = 2.79$; $p = 0.02$; $d = 3.94$, 95% CI = 0.13 – 0.83

*Cluster-corrected with AlphaSim ($p < .05$, minimum $k = 275$). ***p*-values are Bonferroni-corrected for 3 tests; CI were obtained with bootstrapping using 9999 permutations. ASD: Autism spectrum disorder; CI = confidence interval; GL: graphic language; NT: neurotypical; PL: plain language; R: right.

Supplementary references

1 Faul, F., Erdfelder, E., Lang, A.-G. & Buchner, A. G* Power 3: A flexible statistical power analysis program for the social, behavioral, and biomedical sciences. *Behavior research methods* **39**, 175-191 (2007).

2 Baez, S. *et al.* The impact of legal expertise on moral decision-making biases. *Humanities and Social Sciences Communications* **7**, 1-12 (2020).

3 Nasreddine, Z. S. *et al.* The Montreal Cognitive Assessment, MoCA: a brief screening tool for mild cognitive impairment. *Journal of the American Geriatrics Society* **53**, 695-699 (2005).

4 Torralva, T., Roca, M., Gleichgerrcht, E., Lopez, P. & Manes, F. INECO Frontal Screening (IFS): A brief, sensitive, and specific tool to assess executive functions in dementia–ERRATUM. *Journal of the International Neuropsychological Society* **16**, 737-747 (2010).

5 Tsoi, L., Dungan, J. A., Chakroff, A. & Young, L. L. Neural substrates for moral judgments of psychological versus physical harm. *Social cognitive and affective neuroscience* **13**, 460-470 (2018).

6 Hu, Y. *et al.* Right temporoparietal junction underlies avoidance of moral transgression in Autism Spectrum Disorder. *Journal of Neuroscience* **41**, 1699-1715 (2021).

7 Tagliazucchi, E. & Laufs, H. Decoding wakefulness levels from typical fMRI resting-state data reveals reliable drifts between wakefulness and sleep. *Neuron* **82**, 695-708 (2014).

8 Garcia-Cordero, I. *et al.* Metacognition of emotion recognition across neurodegenerative diseases. *Cortex* **137**, 93-107 (2021).

9 Fittipaldi, S. *et al.* A multidimensional and multi-feature framework for cardiac interoception. *NeuroImage* **212**, 116677 (2020).

10 Abrevaya, S. *et al.* At the Heart of Neurological Dimensionality: Cross-Nosological and Multimodal Cardiac Interoceptive Deficits. *Psychosomatic medicine* **82**, 850 (2020).

11 Ibañez, A. *et al.* Predicting and Characterizing Neurodegenerative Subtypes with Multimodal Neurocognitive Signatures of Social and Cognitive Processes. *Journal of Alzheimer's Disease*, 1-22 (2021).

12 Díaz-Rivera, M. N. *et al.* Multidimensional inhibitory signatures of sentential negation in behavioral variant frontotemporal dementia. *Cerebral Cortex* (2022).

13 Birba, A. *et al.* Multimodal Neurocognitive Markers of Naturalistic Discourse Typify Diverse Neurodegenerative Diseases. *Cerebral Cortex* (2021).
